# Advances in Eating Disorders

**DOI:** 10.3390/jcm9124047

**Published:** 2020-12-15

**Authors:** Zaida Agüera, Susana Jiménez-Murcia

**Affiliations:** 1 CIBER Fisiopatología Obesidad y Nutrición (CIBERobn), Instituto de Salud Carlos III, 08907 Barcelona, Spain; 2 Department of Psychiatry, University Hospital of Bellvitge-IDIBELL, 08907 Barcelona, Spain; 3 Department of Public Health, Mental Health and Perinatal Nursing, School of Nursing, University of Barcelona, 08907 Barcelona, Spain; 4 Department of Clinical Sciences, School of Medicine and Health Sciences, University of Barcelona, 08907 Barcelona, Spain

Eating disorders (EDs) are a group of mental disorders characterized by an altered food intake and the presence of inappropriate behaviors for the control of body weight, framed as an excessive concern regarding one’s weight and figure. Anorexia nervosa (AN), bulimia nervosa (BN), binge-eating disorder (BED), and other specified feeding or eating disorders (OSFEDs) are the specific EDs defined in the Diagnostic and Statistical Manual of Mental Disorders, 5th Edition (DSM-5) [1]. All EDs lead to physical and psychosocial functioning impairments in the patients which, in turn, may contribute to the persistence of the disease (for example, the effects of starvation on the brain, social isolation or emotional dysregulation, among others) [2]. Furthermore, the severity of EDs has been highlighted by their chronicity [3], medical complications [4], comorbidity [5]), and the high rates of mortality presented by these patients [6].

To address this important health issue, the current Special Issue collected 21 articles examining the most recent and relevant scientific findings regarding advances in ED. The published articles comprised three reviews and 18 research articles focusing on different aspects, such as genetic [7] and epigenetic factors [8], biomarkers [9], comorbidity [10,11,12,13,14], clinical phenotypes [15,16], neurocognition [12,17,18,19,20,21], treatment predictors [22], and treatment models and therapeutic targets [19,23,24,25,26,27]. Altogether, these studies may provide increased knowledge about the pathogenesis, the risk factors, the maintenance factors, and the most appropriate treatments tools for ED. These articles represented contributions from a diverse group of researchers from multiple countries, including France, Austria, Germany, Belgium, Switzerland, Italy, Spain, the United Kingdom, the United States, Canada, and Japan.

Several relevant findings were observed from this collective body of work. Starting with genetics, Abdulkadir et al. [7] found that genetic factors that underlie body mass index (BMI) are associated with disordered eating behaviors and related cognitions, and that this association is also mediated by BMI. These findings suggest that disordered eating and related cognitions should be considered in a broader context that includes anthropometry as well as behavioral and developmental factors. Regarding epigenetic factors, the review carried out by Steiger and Booij [8] highlights that epigenetic processes link malnutrition and life stresses to risk of ED development. Moreover, differences in the direction of methylation effects have been described in recovered and acute patients with AN. Therefore, authors conclude that DNA methylation could serve as a marker of disease staging or therapeutic response.

The identification of biomarkers was also a relevant topic addressed in this issue. Duriez et al. [9], in a translational study, aimed to determine whether some biomarkers, such as acyl-ghrelin (AG) and desacyl-ghrelin (DAG) plasma concentrations, reflect the level of physical activity during food restriction and after renutrition. Authors found that, in experimental models of mice, AG and DAG both increased during food restriction. They also observed that patients with AN showed a rapid decrease of AG and DAG during weight recovery. However, at one-month post-discharge, AG increased, but only DAG plasma concentrations correlated negatively with BMI and positively with physical activity. Overall, the results highlight the potential role of DAG in the recovery process of AN and suggest a potential negative impact of DAG on weight recovery.

Several included studies also focused on identifying clinical phenotypes in ED. For example, Alvero-Cruz et al. [16] aimed to examine whether somatotype components are predictors of disordered eating in female dance students. The risk of presenting disordered eating was higher in the beginner than in the advanced training group. Authors concluded that somatotype components (ectomorphy and mesomorphy) were predictors of disordered eating in the younger dance student group (beginner training). On the other hand, Matsui et al. [15] focused on identifying the factors related to nocturnal eating syndrome (NES) and sleep-related ED in Japanese young adults. Results showed that the prevalence of NES and sleep-related ED was 2% and almost 5%, respectively. The factors associated with both were smoking, sleepwalking, and use of hypnotic medication. In conclusion, NES, but not sleep-related ED, was associated with a delayed sleep-wake rhythm and sleep disturbances. Meanwhile, Kemmer et al. [22] focused on characterizing physical activity patterns and their effect on weight recovery in patients with AN, and concluded that increased light physical activity in AN decreased after inpatient treatment, and was linked to a greater change in the BMI.

As previously noted, comorbidity is one of the factors that is widely related to the severity and prognosis of ED. In this regard, Buelens et al. [10] investigated whether personality may act as a transdiagnostic mechanism underlying both EDs and non-suicidal self-injury (NSSI) behaviors. The findings of this study revealed an alarmingly high prevalence of NSSI in patients with ED. The patients with ED and NSSI lifetime reported low self-directedness and high harm avoidance, and only those who recently engaged in NSSI showed less novelty seeking. Likewise, Lozano-Madrid et al. [12] aimed to examine the clinical features and neuropsychological performance of ED patients with and without substance use disorder (SUD). Authors found that approximately 19% of patients with ED presented SUD symptoms. Patients with ED and comorbid SUD symptoms displayed a specific phenotype characterized by greater impulsive traits, emotional dysregulation, and more impaired executive control. Testa et al. [11] explored whether attention deficit and hyperactivity disorder (ADHD)-related symptoms may influence the treatment outcome in patients with EDs. Authors found poor treatment outcomes in patients with more severe ED symptoms but an indirect effect of ADHD-related symptomatology. In addition, understanding the role that comorbid autistic features play in patients with AN is also interesting. Therefore, Kerr-Gaffney et al. [13] compared the emotion recognition abilities and attention to faces using eye-tracking in patients with AN and healthy control, concluding that difficulties in emotion recognition appear to be associated with high comorbid autistic traits rather than with a phenotypic feature of AN, independent of illness state. In the same vein, Kinnaird et al. [14] explored the use of a brief sensory sensitivity screener in patients with AN, to assess whether self-rated sensory sensitivity is related to autistic traits. The results of this study showed that patients with AN and high autistic traits scored themselves as more sensitive in the areas of smell, vision, texture, and overall total screening scores, compared to those with low autistic traits.

This collection also includes a set of studies that analyze the role of implicit–explicit emotion in patients with ED and their families, supporting the important role of the emotion recognition [13,14], the expressed emotion [27] or the emotion regulation strategies [12,17] in the development and maintenance of these disorders.

The majority of research of this Special Issue focuses on neurosciences and EDs. There are several studies examining and reviewing a variety of relevant topics such as neuropsychology (especially executive functions) [12,17], structural alterations of the brain [18], neurometabolic alterations [20], neuroimaging studies, and the use and efficacy of invasive and non-invasive brain stimulation techniques [19,21]. Specifically, Mallorquí-Bagué et al. [17] used electrophysiological techniques to explore emotion regulation and food craving regulation in patients with AN. The main findings from this study suggest that patients with AN showed reduced P300 amplitudes and exhibited greater food addiction, emotional dysregulation, and greater use of maladaptive techniques (i.e., suppression strategies) to manage negative emotions than healthy controls. Research with neuroimaging techniques to assess morphological complexity of cortical brain structures from Collantoni et al. [18] provided evidence that cortical fractal dimension (FD) may be a feasible and sensitive method to assess the negative effects of severity and duration of malnutrition in patients with AN. In addition, Maier et al. [20] investigated the metabolic signals in the anterior insular cortex by means of magnetic resonance spectroscopy (MRS). Authors analyzed both acute and recovered patients with AN, as well as healthy controls, and found that metabolic alterations in patients with AN (i.e., lower NAA and Glx signals) seem to be state-related symptoms rather than traits. Villaba-Martínez et al. [19] assessed the efficacy and safety of deep brain stimulation (DBS) applied to chronic, severe, and refractory patients with AN. The findings revealed that DBS was effective for some patients with AN and was associated with self-reported improvements in quality of life. However, almost 40% of the patients presented adverse cutaneous complications. In sum, the review from Duriez et al. [21] concluded that there is not sufficient evidence to support the use of brain stimulation in the treatment of EDs, but that further research on this topic is needed.

Lastly, despite having evidence-based treatments for clinical practice that is well recognized by clinical guidelines, these treatments have proven not to be the panacea. Scientific understanding of the treatment of ED has developed significantly in recent years. Some of these advances in the management of ED have included the complementary use of nutritional protocols, new technologies, brain stimulation techniques, and interventions of support for caregivers of adolescents with ED, among others. For example, Koerner at el. [25] describe the effectiveness and safety of a rapid clinical high-caloric refeeding strategy for patients with AN. Regarding the use of new technologies as an adjuvant therapeutic tool, the findings from Porras-García et al. [24] provide evidence about the usefulness of virtual reality-based body exposure to elicit fear of gaining weight and other body-related disturbances in patients with AN. On the other hand, Philipp et al. [27] demonstrated that parental expressed emotion (associated with higher distress and a lack of skills) was reduced after interventions for caregivers (namely the SUCCEAT program), and that this reduction positively influenced patients’ outcomes. Likewise, the results from the study of Truttmann et al. [26] provide support for the efficacy of the same interventions for caregivers (i.e., SUCCEAT) reducing parental burden, distress and psychopathology. Last but not least, Treasure et al. [23] reviewed the underpinnings of the Cognitive Interpersonal Model for AN and how this can be targeted to treatment. This model encourages the use of treatments targeting changeable elements, such as: increasing social connection and collaborative recovery, using neuromodulation techniques, and augmenting treatment through digital technology.

The purpose of this Special Issue is to address a wide range of topics and contribute to a greater understanding of advances in ED. We believe that the articles contained in the issue have largely achieved this objective. In addition, we would like to thank the various authors and reviewers for their help in amassing this excellent body of work.
